# A rare complication: Infection in acromegalic renal cysts

**DOI:** 10.1002/ccr3.3108

**Published:** 2020-07-16

**Authors:** Yuki Mori, Yuki Otsuka, Yasuhiro Nakano, Hiroyuki Sakae, Kou Hasegawa, Fumio Otsuka

**Affiliations:** ^1^ Department of General Medicine Okayama University Graduate School of Medicine, Dentistry and Pharmaceutical Sciences Okayama Japan; ^2^ Center for Graduate Medical Education Okayama University Hospital Okayama Japan

**Keywords:** acromegaly, renal cyst, urinary tract infection

## Abstract

Renal cysts are detected in one third of acromegaly patients, especially in uncontrolled cases. Clinicians should pay attention to unexpected infection of enlarged renal cysts in acromegaly patients.

## Graphical abstract

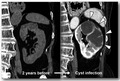

## 1 CASE DESCRIPTION

**Figure 1:**
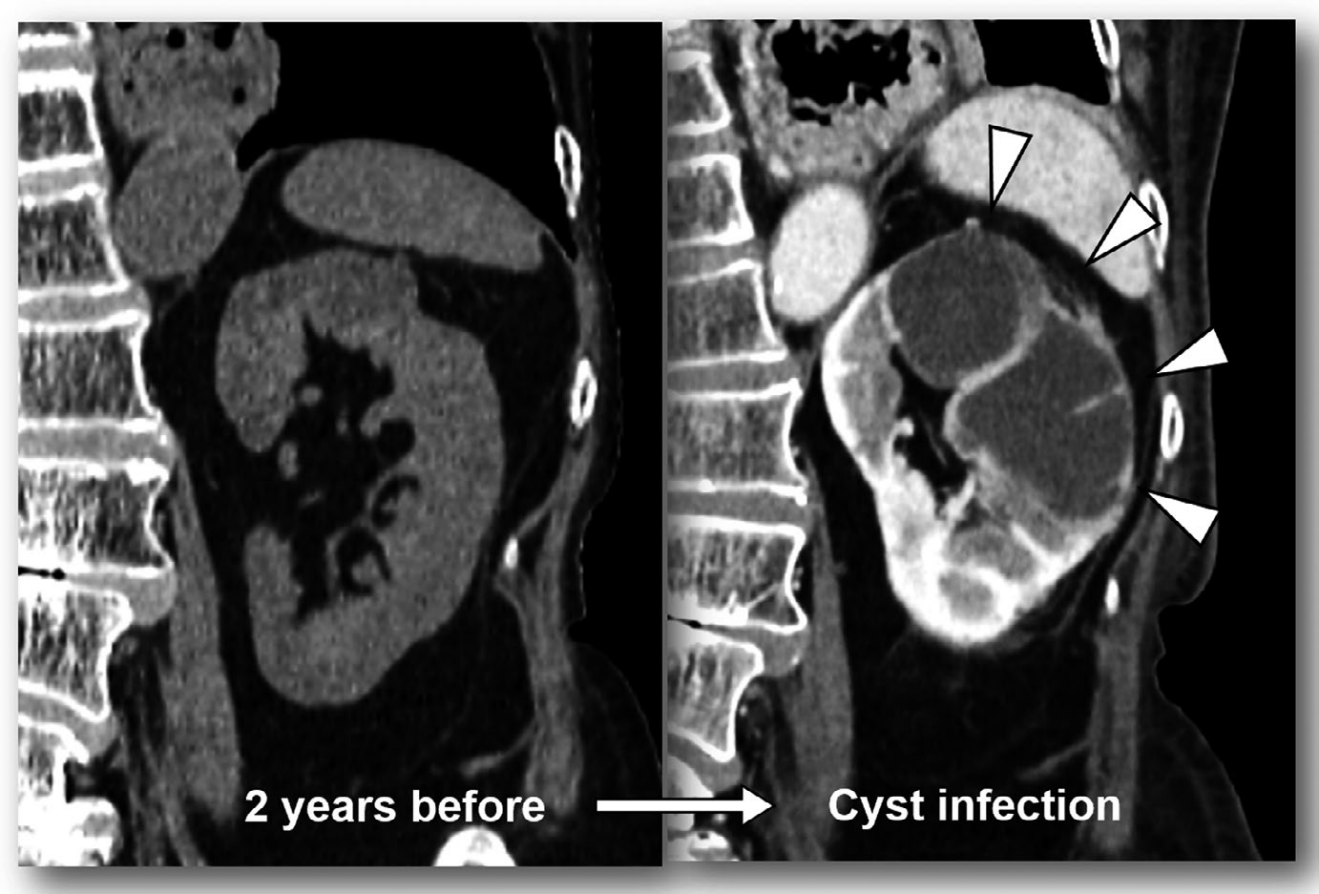
Compared to two years before, enhanced CT revealed that left renal cysts were enlarged with perinephric panniculitis (arrowheads), indicating infectious renal cysts

A 73‐year‐old female with acromegaly who had been treated with octreotide after transsphenoidal surgery 40 years before was referred for fever and malaise. She also had been treated for hypertension, dyslipidemia, osteoporosis, colon polyposis, and diabetes, and all of these were well controlled. She had not developed any renal problems in the past. On physical examination, she had costovertebral angel tenderness, and blood tests showed leukocytosis (14 750/μL) with a high serum level of C‐reactive protein (7.29 mg/dL). Serum growth hormone (GH) and insulin‐like growth factor (IGF)‐I levels were moderately increased to 3.9 ng/mL and 239 ng/mL, respectively. Enhanced CT revealed enlarged left renal cysts with perinephric panniculitis, indicating infectious cysts (Figure 1). *Klebsiella pneumoniae* was detected from a punctatum of the enlarged cysts, and echography‐guided percutaneous drainage with oral levofloxacin was effective.

Acromegaly is often complicated with cystic formation due to excessive GH. Renal cysts are likely to develop in acromegaly, being detected in one third of patients, particularly in elderly patients and patients with a smoking habit who have a high level of GH.^1^ Simple cysts are mostly asymptomatic,however, intractable urinary infection can occur in developed cysts.^2^ Attention must be paid to unexpected infection of acromegalic renal cysts in elderly patients as a rare complication of acromegaly.

## CONFLICT OF INTEREST

None declared.

## AUTHOR CONTRIBUTIONS

YM and YO: wrote the first draft and managed all the submission process. YN, HS, KH, and FO: contributed to the clinical management of the patients and revised the manuscript.
